# Risk prediction models for symptomatic patients with bladder and kidney cancer: a systematic review

**DOI:** 10.3399/BJGP.2021.0319

**Published:** 2021-11-30

**Authors:** Hannah Harrison, Juliet A Usher-Smith, Lanxin Li, Lydia Roberts, Zhiyuan Lin, Rachel E Thompson, Sabrina H Rossi, Grant D Stewart, Fiona M Walter, Simon Griffin, Yin Zhou

**Affiliations:** The Primary Care Unit, Department of Public Health and Primary Care, University of Cambridge, Cambridge.; The Primary Care Unit, Department of Public Health and Primary Care, University of Cambridge, Cambridge.; University of Cambridge School of Clinical Medicine, Addenbrooke’s Hospital, Cambridge.; University of Cambridge School of Clinical Medicine, Addenbrooke’s Hospital, Cambridge.; University of Cambridge School of Clinical Medicine, Addenbrooke’s Hospital, Cambridge.; University of Cambridge School of Clinical Medicine, Addenbrooke’s Hospital, Cambridge.; Department of Surgery, University of Cambridge, Addenbrooke’s Hospital, Cambridge.; Department of Surgery, University of Cambridge, Addenbrooke’s Hospital, Cambridge.; The Primary Care Unit, Department of Public Health and Primary Care, University of Cambridge, Cambridge, and director, Wolfson Institute of Population Health, Queen Mary University of London, London.; The Primary Care Unit, Department of Public Health and Primary Care, University of Cambridge, Cambridge.; The Primary Care Unit, Department of Public Health and Primary Care, University of Cambridge, Cambridge.

**Keywords:** bladder cancer, early diagnosis, kidney cancer, risk prediction, systematic review

## Abstract

**Background:**

Timely diagnosis of bladder and kidney cancer is key to improving clinical outcomes. Given the challenges of early diagnosis, models incorporating clinical symptoms and signs may be helpful to primary care clinicians when triaging at-risk patients.

**Aim:**

To identify and compare published models that use clinical signs and symptoms to predict the risk of undiagnosed prevalent bladder or kidney cancer.

**Design and setting:**

Systematic review.

**Method:**

A search identified primary research reporting or validating models predicting the risk of bladder or kidney cancer in MEDLINE and EMBASE. After screening identified studies for inclusion, data were extracted onto a standardised form. The risk models were classified using TRIPOD guidelines and evaluated using the PROBAST assessment tool.

**Results:**

The search identified 20 661 articles. Twenty studies (29 models) were identified through screening. All the models included haematuria (visible, non-visible, or unspecified), and seven included additional signs and symptoms (such as abdominal pain). The models combined clinical features with other factors (including demographic factors and urinary biomarkers) to predict the risk of undiagnosed prevalent cancer. Several models (*n* = 13) with good discrimination (area under the receiver operating curve >0.8) were identified; however, only eight had been externally validated. All of the studies had either high or unclear risk of bias.

**Conclusion:**

Models were identified that could be used in primary care to guide referrals, with potential to identify lower-risk patients with visible haematuria and to stratify individuals who present with non-visible haematuria. However, before application in general practice, external validations in appropriate populations are required.

## INTRODUCTION

Bladder and kidney cancer are the ninth and 15th most common cancers worldwide, respectively. In the UK, bladder and kidney cancers each account for approximately 3% of new cancer cases, and 5300 and 4500 annual deaths, with their incidence expected to rise.^1^^,^^2^ Early-stage diagnosis is strongly correlated with improved survival rates for both bladder and kidney cancer.^1^^,^^2^

The majority of bladder and kidney cancers (75% and 59%, respectively) are diagnosed following a referral from primary care in the UK.^1^^–^3 A prolonged primary care interval (from patient presentation to referral) is associated with worse clinical outcomes.^4^^,^^5^ Currently, in England, median diagnosis time for bladder and kidney cancer — after presentation in primary care with a relevant clinical feature — is 51 and 70 days, respectively, with variation seen by symptom.^6^

Visible haematuria (VH) is present in the majority of patients with bladder cancer (53%), however, it is less common in individuals diagnosed with kidney cancer (18%).^7^ Currently, the National Institute for Health and Care Excellence guidelines advise urgent referral for suspected bladder or kidney cancer for unexplained non-visible haematuria (NVH) or persistent VH in all individuals aged 60 and 45 years, respectively.^8^ Although 5.1% of people with VH in a primary care setting are ultimately diagnosed with urological cancers, the positive predictive value (PPV) of NVH is less certain and may be as low as 1.6% in primary care.^7^ The focus on haematuria may impede early identification of cancers that present atypically or with a number of non-specific symptoms.^6^^,^^9^ This could also lead to the over-referral of lower-risk individuals presenting with haematuria.^10^

Risk assessment tools have the potential to improve timely diagnosis of cancer by combining multiple clinical features to identify symptomatic patients who would benefit from early referral and reducing investigations in individuals least likely to benefit.^7^^,^^11^ Risk models to guide clinical decision making are becoming more common. For example, the QCancer tool, which estimates the risk of 11 cancers based on symptoms and patient characteristics, has been integrated into primary care software.^12^ Although not routinely used to aid referral decisions for suspected cancer, risk assessment tools have been identified as a potential method for improving UK cancer outcomes.^13^

**Table:** How this fits in

Timely diagnosis of bladder and kidney cancer from primary care is key to improving survival rates, but remains challenging. Risk models have been suggested as a possible tool to guide clinicians in making referral decisions, particularly in individuals who present atypically. This systematic review identified a number of models that may be of interest, in particular, models able to identify low-risk individuals who may not require referral and a model suitable for stratifying risk in individuals with non-visible haematuria. However, only a small number of models included clinical features other than haematuria and there was a lack of external validations.

In this review, published models that incorporate symptoms and signs (referred to as clinical features) and estimate the risk of undiagnosed prevalent bladder or kidney cancer at an individual level were systematically identified and compared. The review focuses on the risk factors included in the models, the performance of the models (discrimination and calibration), and their potential use in primary care.

## METHOD

A systematic review was performed following an a priori established study protocol (PROSPERO ID: CRD42018116967).

An electronic literature search of MEDLINE and EMBASE was performed in November 2018 and updated in December 2020. Literature published 1980–2020 was included, using a combination of subject headings incorporating ‘bladder *or* renal/kidney *or* urinary-tract cancer’, ‘risk *or* risk factor *or* chance’ *and* ‘model *or* prediction *or* score’ (see Supplementary Table S1 and S2).

Studies were included that fulfil all of the following criteria:

- are published, peer-reviewed, primary research;
- present a model, which here is considered the use of a combination of ≥2 factors to identify individuals with a higher risk of undiagnosed prevalent bladder or kidney cancer. Studies predicting recurrent or future risk were excluded;
- incorporate at least one clinical feature as a risk factor;
- include at least one quantitative measure of model performance (discrimination, calibration, or accuracy). Accepted measures include (but are not limited to) area under the receiver operating curve (AUROC), *R*^2^ (goodness of fit), sensitivity, specificity, PPVs, and negative predictive values (NPVs). Graphical measures alone were not accepted; and
- are applicable to the general population. Studies including only specific groups — for example, individuals receiving dialysis — were excluded.

One reviewer carried out the search. Reviewers screened titles and abstracts to exclude clearly irrelevant articles. Pilot screening was carried out to ensure consistency between reviewers. The full text was examined, by two reviewers, if a definite decision to exclude could not be made based on the title and abstract alone. Disagreements were resolved by discussion with a third reviewer.

Data extraction was carried out independently by two reviewers for all included studies. Where studies included multiple different models all were included separately. Details of model development, validation, and performance were extracted into a standardised form. Included studies were classified according to the TRIPOD guidelines.^14^ The PROBAST tool was used to assess risk of bias (RoB) over four domains of interest (population, risk factors, outcomes, and analysis).^15^^,^^16^ Information required for this assessment was extracted by two reviewers, and one reviewer scored the studies. A second reviewer checked the RoB assessment process.

## RESULTS

**Figure 1.**
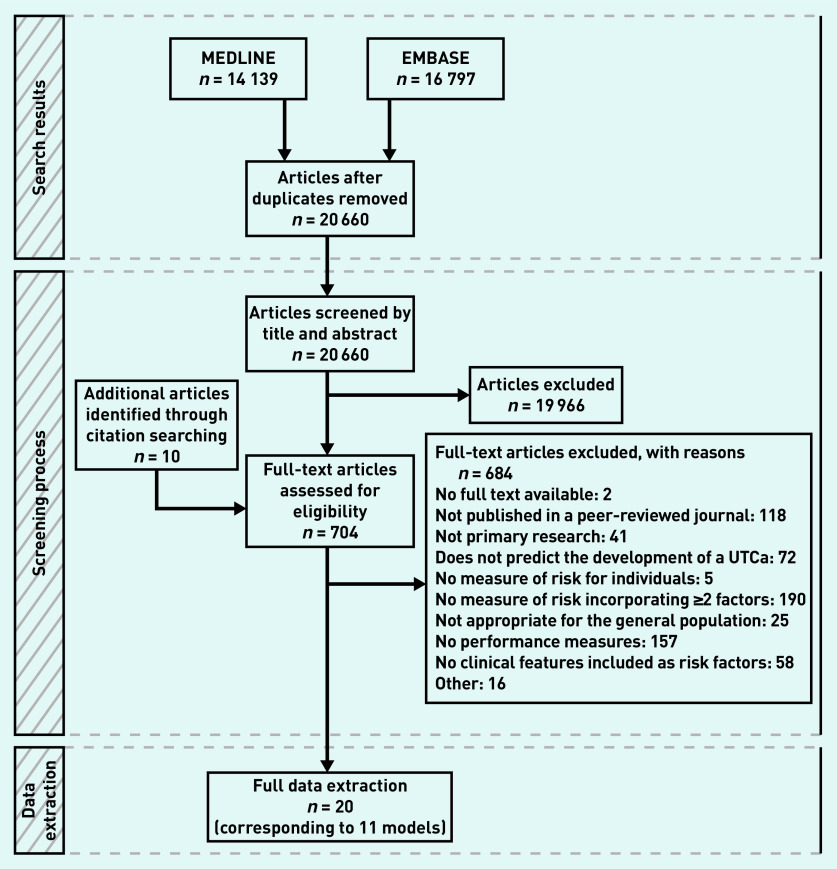
*PRISMA flow diagram. UTCa = urinary tract cancer.*

After duplicates were removed, the search identified 20 661 articles. Of these, 19 959 were excluded by title and abstract screening, and 686 after full-text assessment. Twenty studies were identified, describing 29 models that satisfied the inclusion criteria (Figure 1).^10^^,^^17^^–^35

### Study design and setting

Of the 20 studies, 16 were cohort studies^10^^,^^17^^,^^19^^–^30^,^^33^^,^^34^ and four were case–control studies^18^^,^^31^^,^^32^^,^^35^ (see Supplementary Table S3). Six studies were performed in a UK primary care setting, using routinely coded data.^22^^–^24^,^^31^^,^^32^^,^^35^ Nine were conducted in secondary (or specialist) care settings, including hospital outpatient clinics and urology departments.^10^^,^^18^^,^^21^^,^^26^^–^28^,^^30^^,^^33^^,^^34^ The remaining five studies do not provide enough information about the study setting to be classified as primary or secondary^17^^,^^19^^,^^20^^,^^25^^,^^29^ (for example, referring to recruitment at a ‘clinic’).

Most studies included European (*n* = 11) or North American populations (*n* = 8); two studies were based in South East Asia.^21^^,^^26^ The six studies in a primary care setting included a mixture of asymptomatic and symptomatic individuals.^22^^–^24^,^^31^^,^^32^^,^^35^ Eleven studies included patients undergoing investigation for haematuria,^10^^,^^18^^–^21^,^^26^^,^^27^^,^^28^^,^^30^^,^^33^^,^^34^ in some cases restricted to NVH (*n* = 2)^27^^,^^34^ or painless haematuria (*n* = 4).^18^^–^20^,^^26^ Three studies included individuals classified as high-risk based on a prior history of haematuria^30^ or smoking status.^17^^,^^29^ One study included all individuals enrolled on a health insurance plan who underwent urinalysis.^25^

Of the 29 models (Table 1), the outcomes were a diagnosis of bladder cancer (*n* = 19),^17^^–^21^,^^29^^,^^32^^–^35 kidney cancer (*n* = 1),^31^ or urological cancer (*n* = 9) (bladder and kidney cancer, either with^10^^,^^22^^,^^25^ or without^23^^,^^24^^,^^27^^,^^28^ cancers of the urothelium). Most models were developed in mixed-sex populations, although a small number were developed specifically for males (*n* = 2)^22^^,^^23^ and females (*n* = 2).^22^^,^^24^ The majority of the models were developed using logistic regression (*n* = 22), although other methodologies, including survival models (*n* = 2), were also found. Internal validation — either bootstrapping^17^^–^20 or split-sampling (random^22^^–^24^,^^34^ or nonrandom^28^^,^^29^) — has been carried out for 22 models. Only eight models have been externally validated.^10^^,^^25^^,^^27^^,^^28^

**Table 1.** Summary of included models

**Model^a^ (author [ year],^ref^ model)**	**Outcome**	**Summary of risk factors**								**Validation^b^**	**Development setting**
		**Demographic and lifestyle**			**Clinical symptoms and signs**				**Test results**		
		**Age**	**Sex**	**Smoking**	**Other**	**Haematuria**	**System specific**	**Non-specific**			
Hippisley-Cox (2012),^22^ a	KCa, BCa, UCa	—	—	x	x	VH	Abdominal pain	Appetite loss, weight loss, anaemia	—	Internally	P
Hippisley-Cox (2012),^22^ b	KCa, BCa, UCa	—	—	x	x	VH	Abdominal pain	Appetite loss, weight loss, anaemia	—	Internally	P
Hippisley- Cox 2013),^23^ a	KCa, BCa	x	—	x	x	Unspecified	Abdominal pain	Night sweats, weight loss	—	Internally	P
Hippisley- Cox (2013),^24^ b	KCa, BCa	x	—	x	x	Unspecified	Abdominal pain, postmenopausal bleeding	Anaemia, appetite loss, indigestion, weight loss	—	Internally	P
Shephard (2013),^31^ a	KCa	x	—	—	—	VH	Back pain, abdominal pain, UTI	Fatigue, constipation, nausea	x	None	P
Shephard (2012)^32^	BCa	x	—	—	—	VH	Dysuria, abdominal pain, UTI	—	x	None	P
Price (2014)^35^	BCa	x	—	—	—	VH and NVH	Dysuria, abdominal pain, UTI	Constipation	x	None	P
Jung (2011),^25^ a	KCa, BCa, UCa	x	—	—	—	VH or NVH	—	—	—	Externally	U
Jung (2011),^25^ b	KCa, BCa, UCa	x	—	—	—	NVH	—	—	—	Externally	U
Loo (2013),^28^ a	KCa, Bca	x	x	x	—	NVH and history	—	—	—	Externally	S
Loo (2013),^28^ b	KCa, Bca	x	x	x	—	NVH and history	—	—	—	Externally	S
Beukers (2013),^18^ a	BCa	x	x	—	—	Degree	—	—	x	Internally	S
Beukers (2013),^18^ b	BCa	x	x	—	—	Degree	—	—	x	Internally	S
Cha (2012),^19^ a	BCa	x	x	x	—	Degree	—	—	—	Internally	U
Cha (2012),^19^ b	BCa	x	x	x	—	Degree	—	—	x	Internally	U
Cha (2012),^19^ c	BCa	x	x	x	—	Degree	—	—	x	Internally	U
Cha (2012),^19^ d	BCa	x	x	x	—	Degree	—	—	x	Internally	U
Hee (2013)^21^	BCa	x	x	x	—	Degree	—	—	—	Externally	S
Lotan (2009),^29^ a	BCa	x	x	x	x	Degree	—	—	—	Internally	U
Lotan (2009),^29^ b	BCa	x	x	x	x	Degree	—	—	x	Internally	U
Lotan (2009),^29^ c	BCa	x	x	x	x	Degree	—	—	x	Externally	U
Lotan (2009),^29^ d	BCa	x	x	x	x	Degree	—	—	x	Internally	U
Barbieri (2012),^17^ a	BCa	x	x	x	x	Unspecified	—	—	—	None	U
Barbieri (2012),^17^ b	BCa	x	x	x	x	Unspecified	—	—	x	None	U
Barbieri (2012),^17^ c	BCa	x	x	x	x	Unspecified	—	—	x	None	U
Barbieri (2012),^17^ d	BCa	x	x	x	x	Unspecified	—	—	x	None	U
Tan (2019)^33^	BCa	x	x	x	—	Degree	—	—	—	Externally	S
Georgieva (2019)^10^	KCa, BCa, UCa	x	x	x	—	History	—	—	—	Externally	S
Matulewicz (2020)^34^	BCa	x	x	x	x	NVH	—	—	—	—	S

a *a, b, c refers to models developed by the same author group.*

b *Validation: none — development only; internally — at least internally validated; and externally — externally validated. BCa = bladder cancer. degree = visible haematuria or non-visible haematuria. KCa = kidney cancer. NVH = non-visible (microscopic) haematuria. P = primary care. S = secondary care. U = unknown. UCa = cancer of the ureter. UTI = urinary tract infection. VH = visible (gross) haematuria. x = included in the model.*

### Risk factors

Haematuria was included as a risk factor in all of the included models (see Supplementary Table S4). However, there was significant variation in the type of haematuria included. Four models used only VH,^22^^,^^31^^,^^32^ four only NVH,^25^^,^^28^^,^^34^ and 14 included both (as separate risk factors [ *n* = 2]^25^^,^^35^ or the degree of haematuria was used as a risk factor [ *n* = 12]^18^^–^21^,^^29^^,^^33^). In seven models, the type of haematuria was unspecified.^17^^,^^23^^,^^24^

Most studies (*n* = 14) reported the association between haematuria and the outcome of interest (see Supplementary Table S5). Frequently the presence of haematuria, either any (*n* = 3), visible (*n* = 5), or non-visible (*n* = 2), was compared with no haematuria. One study reported the odds ratio (OR) separately for both VH (OR 26, 95% confidence interval [CI] = 22 to 30) and NVH (OR 20, 95% CI = 12 to 33) for bladder cancer.^35^ Four studies, developed in cohorts composed of individuals undergoing investigation for haematuria for suspected bladder cancer, gave ORs for individuals with VH compared with those with NVH.^18^^,^^20^^,^^21^^,^^33^ All showed stronger associations with VH than NVH (OR 1.71–3.85 in multivariate analysis).

Seven models included other clinical features in addition to haematuria.^22^^–^24^,^^31^^,^^32^^,^^35^ These included abdominal pain (*n* = 7), weight loss (*n* = 4), anaemia (*n* = 3), loss of appetite (*n* = 3), urinary tract infection (UTI) (*n* = 3), and dysuria (*n* = 3). In each case, the risk because of haematuria was at least eight times higher than the risk from all other clinical features.

Demographic risk factors, including age (*n* = 27), sex (*n* = 20), and ethnic group (*n* = 9), were used in most models. Modifiable lifestyle risk factors, including smoking (*n* = 24) and BMI (*n* = 2), were also considered. Three models included abnormal blood tests;^31^^,^^32^ eight urine biomarkers^17^^,^^18^^,^^20^^,^^29^ and seven urine cytology.^17^^,^^18^^,^^20^^,^^29^

### RoB

**Figure 2.**
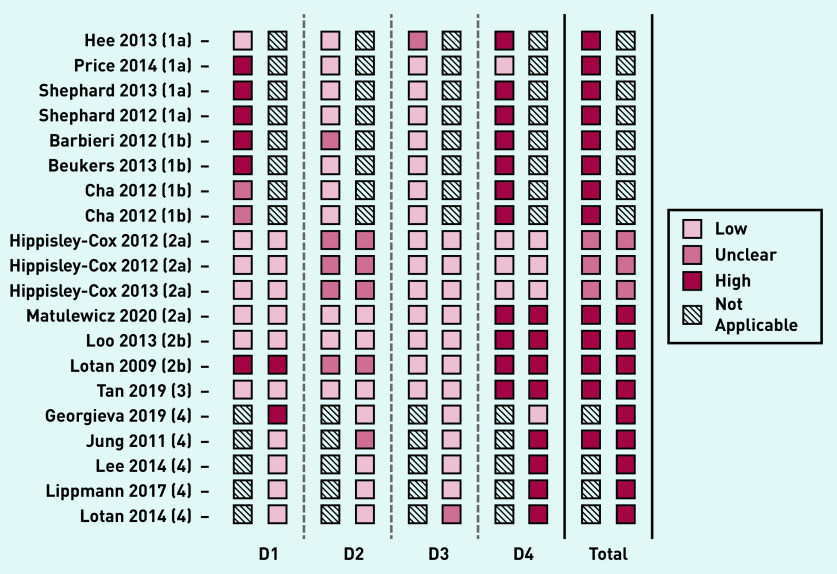
***RoB assessment using PROBAST framework.*** *^a^* *^a^****For each study, RoB is shown for model development and validation separately. RoB is assessed over four domains (D1: population, D2: risk factors, D3: outcome, D4: analysis), the overall results for each study are shown on the right. 1, 2, 3, and 4 refer to TRIPOD classification for each identified study.****14* ***a, b, and c refer to models developed by the same author group. RoB = risk of bias.***

Most of the 20 studies included in this systematic review were assessed to have a high RoB (*n* = 17) in both development and validation (Figure 2). The most common issues were seen in domain 4 (analysis), in which 11 of 15 development studies and eight of 12 validation studies were at high RoB. This was frequently because of an insufficient number of cases or incomplete reporting of performance measures (including not reporting calibration of model).

### Performance measures

**Figure 3.**
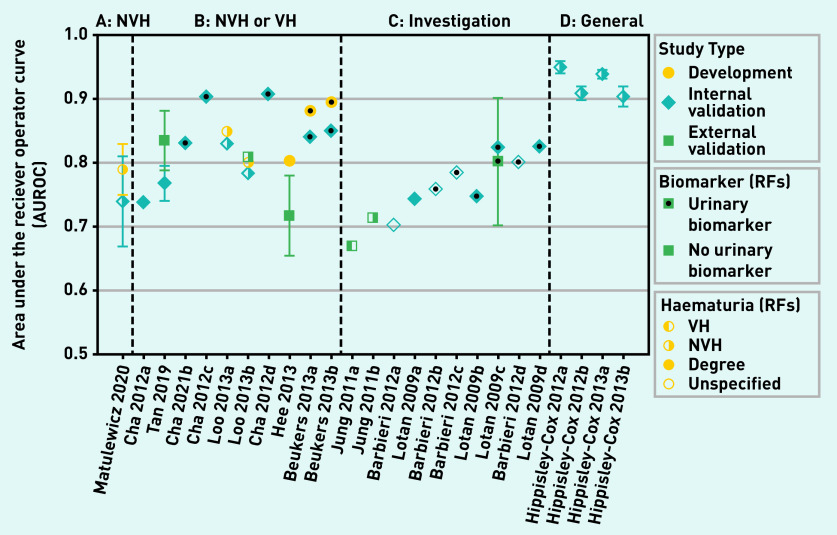
***Model discrimination, AUROC.*** *^a^* *^a^* ***Models are split into groups describing the development population and within each group are ordered by the number of risk factors used. Study type (development, internal and external validation), type of haematuria used in model, and study setting are indicated on the plot. A, b, and c refer to models developed by the same author group. Each model is labelled according to its development study; however, the discrimination measured in several external validations*** *26 ^,^ 27 ^,^ 30* ***of these models are also included in this summary plot (see supplementary Table S6 for details). AUROC = area under the receiver operating curve. NVH = non-visible haematuria. RF = risk factor. VH = visible haematuria.***

Discrimination (the AUROC) was reported for 26 models (Figure 3 and Supplementary Table S6). Calibration was reported for 13 internal^20^^,^^22^^–^24^,^^29^^,^^34^ and three external validations.^21^^,^^30^^,^^33^

The four Hippisley-Cox and Coupland models, developed in unfiltered population-based cohorts to predict urological cancer, all have AUROC values in the range 0.88–0.96 in a large internal validation (Figure 3, group D).^22^^–^24 These models report good calibration and relatively high levels of accuracy (sensitivity 0.77–0.71, specificity 0.90–0.91) when using the 90th percentile of risk as a cut-off. They also have high NPV (100%) for PPVs in the range 0.6%–1.6% for this threshold. The two models developed for males have slightly higher discrimination than those for females. Demographic and lifestyle risk factors are combined with clinical features — smoking, haematuria, and abdominal pain feature in all four. Two specified VH as a symptom, whereas the other two did not specify type of haematuria. This did not significantly affect performance; however, other risk factors also differed between these models.

The models by Shephard *et al* and Price *et al* predicted the risk of developing kidney^31^ and bladder^32^^,^^35^ cancer by combining pairs of symptoms observed in unfiltered population-based cohorts. The combinations of symptoms with the highest accuracy were microcytosis and abdominal pain for kidney cancer (PPV >5%), and VH and raised white blood cell count for bladder cancer (PPV 8.8%). It is shown^35^ that, even in older age groups (>60 years), the PPV of NVH for bladder cancer is low (0.8%), however, when combined with dysuria, for example, this increases to 4.5%. These symptom combinations are rare (<10 cases out of 3140 in development population), so may have limited impact individually.

The model by Matulewicz *et al*^34^ was developed in a population with newly diagnosed NVH and had an AUROC value of 0.74 (95% CI = 0.67 to 0.80) in an internal validation (Figure 3, group A). This model combines a categorical measurement of NVH (red blood cells per high-power field [RBC/hpf]) with age, sex, smoking, and ethnic group to predict a likelihood of a bladder cancer diagnosis. For a threshold (>5% risk) that gives a PPV of 10.4%, reasonable accuracy (sensitivity 68%, specificity 75%) and a high NPV (98%) are demonstrated.

The remaining 20 models report discrimination in populations undergoing investigation for suspected urological cancer, with varying proportions of the populations having VH and NVH (Figure 3, groups B and C). On average, discrimination is higher in models developed only in individuals with haematuria (group B) and in models that incorporate urinary biomarkers. The model with highest discrimination in external validation was Tan *et al* (2019) (AUROC = 0.77).^33^ This model combines type of haematuria with age, sex, and smoking status to predict the risk of a bladder cancer diagnosis. For an optimised cut-off point (>4.015%), the reported accuracy measures indicate high sensitivity (0.99) can be achieved; however, the corresponding specificity was low (0.31). The best performing models incorporating urinary biomarkers are Cha *et al* (2012) model c and Cha *et al* (2012) model d (AUROC = 0.9 in internal validation).^19^ The degree of haematuria (VH or NVH) is combined with the uCyt assay (an immunocytochemical test that detects markers from malignant urothelial cells in urine)^36^ and several demographic and lifestyle factors. Cha *et al* (2012) model d also included the results of cytology as a risk factor; this does not seem to improve model performance. The models by Loo *et al* 28 include an indication of the severity of NVH (>25 RBC/hpf); Loo *et al* (2013) model b has high discrimination (AUROC = 0.809) in external validation.^27^

## DISCUSSION

### Summary

This review found 13 risk prediction models with good discrimination (>0.8) for urological cancer. All of the models included haematuria and seven incorporated additional clinical signs or symptoms. Most were developed in populations undergoing investigation for suspected urological cancer, with only seven developed in primary care (or unfiltered population-based) cohorts. Only eight of the identified models had been externally validated and around half (*n* = 14) had no reported measure of calibration.

### Strengths and limitations

This is the first study, to the authors’ knowledge, to provide a systematic and up-to-date review of the existing risk prediction models for bladder or kidney cancer with application to primary care. The study benefits from a comprehensive search and rigorous screening of studies for inclusion. In total, 29 models were identified in this process, providing a clear overview of the current research in this area. The PROBAST tool was used, a new quality assessment tool for risk prediction models, to perform a robust assessment of the RoB for each model and identify areas where the quality of research is low. It was not possible to perform a meta-analysis because of the heterogeneity in the study designs, including differences in study type (development and validation), design (cohort and case–control), setting (primary and secondary care), and recruitment criteria. A further limitation is that several models used coded information from primary care records and may be subject to bias in clinician recording and choice of investigations.

### Comparison with existing literature

Recent reviews have examined risk assessment tools for the identification of other undiagnosed cancers, including colorectal^37^ and ovarian cancer.^38^ The models identified by those studies had similar discriminative ability to those described in this review. As in this review, a lack of high-quality studies and external validations was noted. There was a wider range of models developed specifically for primary care settings for those cancers, than have been identified in the current study for urological cancer.

Although VH has been widely shown to be associated with urological cancer,^39^ the association with other clinical factors (including NVH and UTIs) is poorly understood,^7^^,^^39^ with variation between different populations.^40^ In this review, only seven models included clinical factors other than haematuria and only five studies directly compared VH and NVH as risk factors. Additionally, haematuria has a much higher contribution than other clinical risk factors in all models where >1 is used.

### Implications for research and practice

The seven models developed in primary care settings^22^^–^24^,^^31^^,^^32^^,^^35^ are the most applicable to this review question. The excellent performance of the four Hippisley-Cox models, if replicated in an external validation, would make them suitable for use in primary care, in particular, they may enable clinicians to identify lower-risk individuals who do not need referral. However, it is unclear how these models would be used and how this would compare with current practice. For example, it cannot be inferred if any individuals currently eligible for referral (such as those with VH) would be reclassified using these models.

The model developed by Matulewizc,^34^ in a population with newly identified NVH, could be used in primary care to guide referral decisions in individuals with NVH. Current guidelines for referral for suspected urological cancer in the UK differentiate between types of haematuria (VH and NVH) and age (>45 and >60 years, respectively). There is concern that lower-risk patients, such as younger individuals with NVH, are not managed optimally.^7^ The Matulewizc model, by combining a categorical measure of NVH with demographic factors, identifies both high- and low-risk individuals successfully (PPV 10.4% and NPV 98.2%). This suggests that this model could identify some individuals with NVH who are aged <60 years who would benefit from referral and some aged >60 years who are at lower risk and do not need referral. The high PPVs seen when using this model, and when NVH was combined with other clinical signs in the study by Price *et al*,^35^ indicate the need to consider the broader clinical context when making referral decisions in patients with NVH.

In conclusion, haematuria was the strongest clinical risk factor associated with urological cancers and was included in all of the models identified. Several models have been developed in primary care populations that could be used to guide referrals, in particular, identifying those at lower risk least likely to benefit from further investigation. Additionally, one model was identified that could be used to stratify the risk of cancer in individuals presenting with NVH.

Future research in this area should initially focus on carrying out external validations of the identified models in a suitable primary care cohort. Researchers should then consider the impact that implementing these models to support referral decisions would have on both patient outcomes and the healthcare service in their analyses.

## Data

Templates of data extraction forms and code used to produce graphs are not publicly available, but will be provided on contacting the corresponding author.
